# Acoustic extraordinary transmission manipulation based on proximity effects of heterojunctions

**DOI:** 10.1038/s41598-018-37724-y

**Published:** 2019-01-31

**Authors:** Zhi-Yong Tao, Ting Liu, Chuan Zhang, Ya-Xian Fan

**Affiliations:** 1 Key Lab of In-fiber Integrated Optics, Ministry Education of China, Harbin Engineering University, Harbin, 150001 People’s Republic of China; 2 Academy of Marine Information Technology, Guilin University of Electronic Technology, Beihai, 536000 People’s Republic of China; 3 Physics Research Centre, College of Science, Harbin Engineering University, Harbin, 150001 People’s Republic of China

## Abstract

Heterojunctions between two crystalline semiconductor layers or regions can always lead to engineering the electronic energy bands in various devices, including transistors, solar cells, lasers, and organic electronic devices. The performance of these heterojunction devices depends crucially on the band alignments and their bending at the interfaces, which have been investigated for years according to Anderson’s rule, Schottky-Mott rule, Lindhard theory, quantum capacitance, and so on. Here, we demonstrate that by engineering two different acoustic waveguides with forbidden bands, one can achieve an acoustic heterojunction with an extraordinary transmission peak arising in the middle of the former gaps. We experimentally reveal that such a transmission is spatially dependent and disappears for a special junction structure. The junction proximity effect has been realized by manipulating the acoustic impedance ratios, which have been proven to be related to the geometrical (Zak) phases of the bulk bands. Acoustic heterojunctions bring the concepts of quantum physics into the classical waves and the macroscopic scale, opening up the investigations of phononic, photonic, and microwave innovation devices.

## Introduction

Heterojunctions refer to the interface regions formed by the contact of two different semiconductors^1–5^. Semiconductor heterojunctions have many significant properties, such as quantum effects, mobility increase characteristics, singular second-degree spatial properties, and intensity interface states^6–8^, which provide advantages to engineer the electronic and photonic devices, such as transistors^9^, solar cells^10^, semiconductor lasers^11^, and organic electronic devices^12^. It has been found that the junction properties highly rely on energy band alignments at the interface, where the energy offset of band structures changes due to the space charge effects^13^. The changes known as band bending have been successively investigated by Schottky, Mott, Anderson, Bardeen, Tersoff, and so on^14^. Based on the works of these eminent scientists, heterojunctions have been extensively studied and used in such as thermionic emission^15^, photovoltaic^16,17^, polariton^18^, band engineering^19,20^, and controllable spontaneous emission^21^ devices.

In recent twenty years, due to the similar band structures as semiconductor materials, the artificial periodic structures, known as metamaterials, have also attracted a rapidly growing interest, such as sonic crystals^22^, superlens^23,24^, negative refraction^25,26^, electromagnetic cloaks^27^, thermal diodes^28^, and acoustic topological materials^29–33^. As with their semiconductor counterparts, integrating metamaterials with different band gaps can result in heterojunctions at interfaces, which have already been used in nanophotonics to achieve high performance devices^34^ and all-optical memory^35^. However, the performance of heterojunctions is still a complex problem due to its dependence on junction structures, which play an essential role in electronic transport properties of semiconductors and have been known as contact problems^14^, proximity effects^36^, and geometric effects^37^. Lacking the understanding of junction proximity effects makes us lose our chances to achieve the desired devices and to control or tune their performances. Recently, the topological phase transition has been applied to the silica based phononic crystals^38^ and the nanophononic system^39^, in which the topological interface states have been observed. Especially, Esmann *et al*. has paid attention to the finite size effects in their appendix^39^, which cannot be included in the topological theories.

In this work, we designed and fabricated the acoustic waveguides with different heterojunctions to experimentally and theoretically investigate the junction proximity effects in the macroscopic scale. Mimicking semiconductor heterojunctions, we connected two periodically corrugated acoustic waveguides with different band gaps and unexpectedly found a transmitted peak in the former gap. Taking the advantage of acoustic scales, we elaborated two series of acoustic junctions by shifting the phases of two duct ends and experimentally demonstrated the spatial dependences of the transmission on heterojunctions. The proposed initial phase of wall corrugations is more convenient for wave control engineering than the topological phases defined in momentum space. The further theoretical and numerical results revealed the mechanism of the junction proximity effects, which would benefit the underlying physics exploration on heterojunction geometries and pave the way for quantum and classical functional devices based on heterojunctions.

## Results

### Acoustic heterojunctions

**Figure 1:**
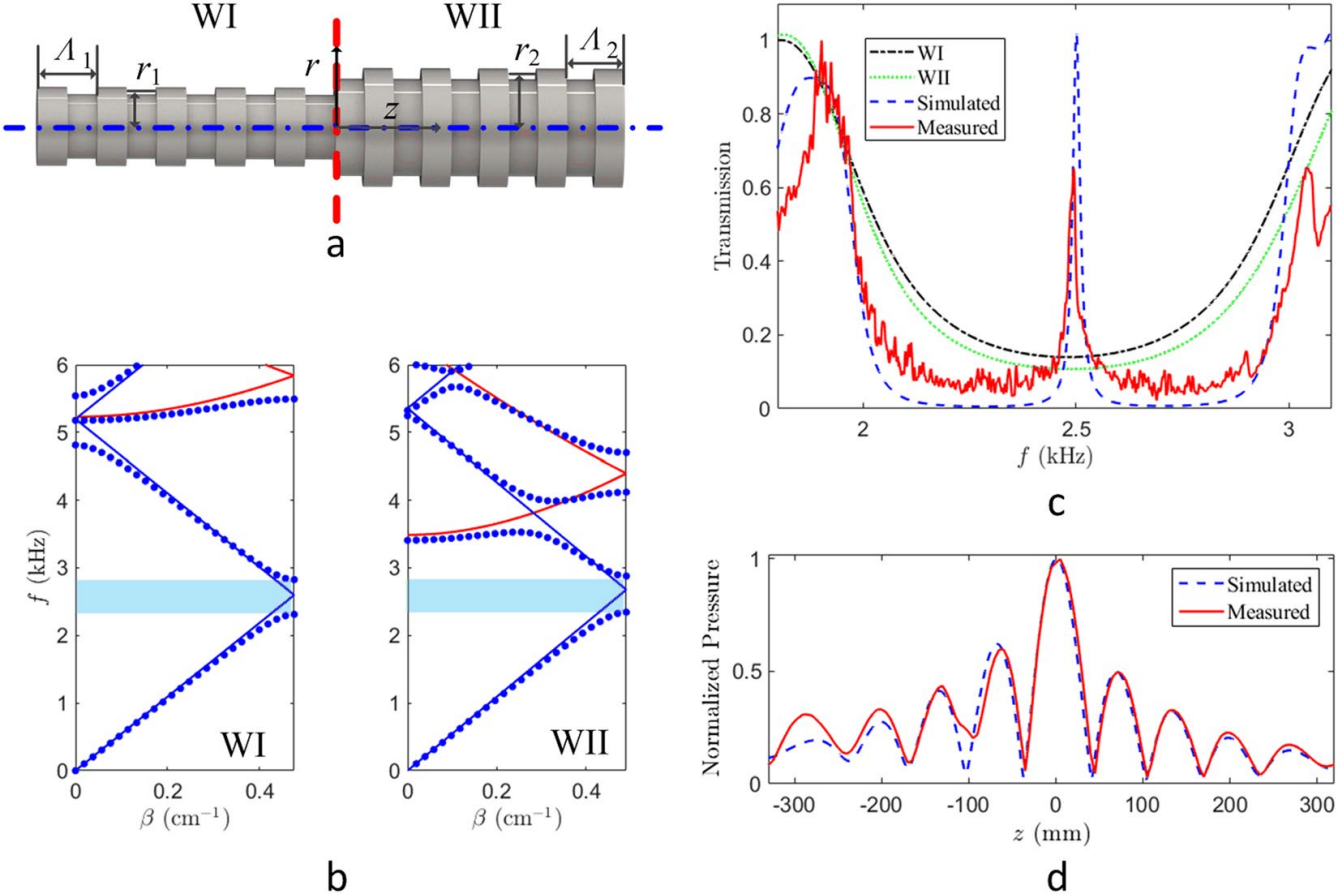
Acoustic heterojunction and its extraordinary transparency. (**a**) Acoustic heterojunction waveguide consisting of two ducts with different geometry parameters. The average radii of Waveguide I (WI) and II (WII) are *r*_1_ and *r*_2_, respectively, and the periods are *Λ*_1_ and *Λ*_2_. The red dashed line marks the acoustic heterojunction while the blue dash-dot line shows the symmetric axis of the waveguide. (**b**) Band alignment of two waveguides with different geometry parameters but the similar Bragg band gap marked by the blue shadows. The blue dots depict the calculated dispersion curves while the blue and red solid lines denote the fundamental and first transverse modes, respectively. (**c**) Transmission of acoustic waveguides. The red solid line shows the experimentally measured data, where an extraordinary transparency arising in the Bragg gap. As the references, the simulated data are shown by the blue dashed line for the heterojunction waveguide, and by the black dash-dot and green dotted lines, respectively, for WI and WII. (**d**) Normalized sound pressure along the symmetry axis. Both the measured (the red solid line) and the simulated (the blue dashed line) pressures exhibit the sound energy localization at the heterojunction.

The proposed heterojunction waveguide is composed of two corrugated ducts with different parameters as shown in Fig. 1a. To mimic the heterojunctions, the period and average radius of Waveguide I (WI) are selected as *Λ*_1_ = 66 mm and *r*_1_ = 40 mm, respectively, while these of Waveguide II (WII) are *Λ*_2_ = 64 mm and *r*_2_ = 60 mm. Both the corrugation amplitudes are selected as ten percent of their own average radii. Thus, the wide and narrow radii of WI are 44 mm and 36 mm, respectively, whereas these of WII are 66 mm and 54 mm. The blue dash-dot line denotes the symmetry axis and the red dashed line marks the junction interface between two waveguides. *r* and *z* are the cylindrical coordinates for radius and longitudinal directions as shown in Fig. 1a, respectively.

The band structures of WI and WII can be estimated by the reference lines^40^,

1 $$f=\frac{c}{2\pi }\sqrt{\frac{{k}_{r,m}^{2}}{{r}_{0}^{2}}+{(\frac{2n\pi }{{\Lambda }}+\beta )}^{2}},$$

where *f* and *c* are the frequency and speed of sound, respectively. *k*_*r,m*_ is a zero of the first-order Bessel function, *β* is the propagation constant (−*π*/*Λ* ≤ *β* ≤ *π*/*Λ*). *r*_0_ and *Λ* are the average radius and period of the waveguide, respectively. The blue and red solid lines in Fig. 1b respectively denote the fundamental (*m* = 0) and first (*m* = 1) transverse modes of the waveguides. The Bragg resonances will occur at the intersections of the blue solid lines and create the Bragg band gaps. The calculated dispersion curves for the two waveguides also confirm the similar forbidden band creations as shown in Fig. 1b by the light blue shadows. The band alignment of these two waveguides with different geometry parameters suggests the formation of an acoustic heterojunction, while it would be very interesting to bring the semiconductor concepts into such a classical system.

With COMSOL Multiphysics, we have calculated the sound transmission through the waveguide around the Bragg gap. The transmission is defined by the sound energy ratio of the outlet and inlet. The black dash-dot and green dotted lines in Fig. 1c present the energy transmissions of WI and WII, respectively, indicating that these two waveguides are opaque to the sound in the frequency range of 2–3 kHz. However, in the experiment, we have observed an extraordinary transparency with very sharp peak at 2.5 kHz, depicted by the red solid line in Fig. 1c. The 2.5 kHz transmitted peak is outstanding in the forbidden band, illustrating the heterojunction effects on the acoustic wave transmission. As a reference, the simulated transmission is also depicted by the blue dashed line in Fig. 1c. Both the measured and simulated results confirm the presence of the extraordinary transparency, which provides the convincing evidence for the heterojunction effects on the classical waves.

In addition, we have experimentally recorded the sound pressure along the symmetry axis of the waveguide and present the normalized one in Fig. 1d by the red solid line, which is also accompanied by the numerical results in the blue dashed line. Most of numerical and experimental results are in good agreement except a little deviation caused by the slight machining imperfections. From the sound pressure distributions, we have found the sound pressure localization at the junction interface. It tells us that the acoustic heterojunction not only brings us an exterior extraordinary transparency but also provides the physical mechanism of an interface state with energy localization at the junction. In contrast to quantum systems, heterojunctions prefer to raise an interface state which localizes the energy at the junction for classical waves, but the junction structures still have effects on the transparency as the widely considered contact problems, proximity effects, and geometric effects in semiconductor physics.

### Junction proximity effects

**Figure 2:**
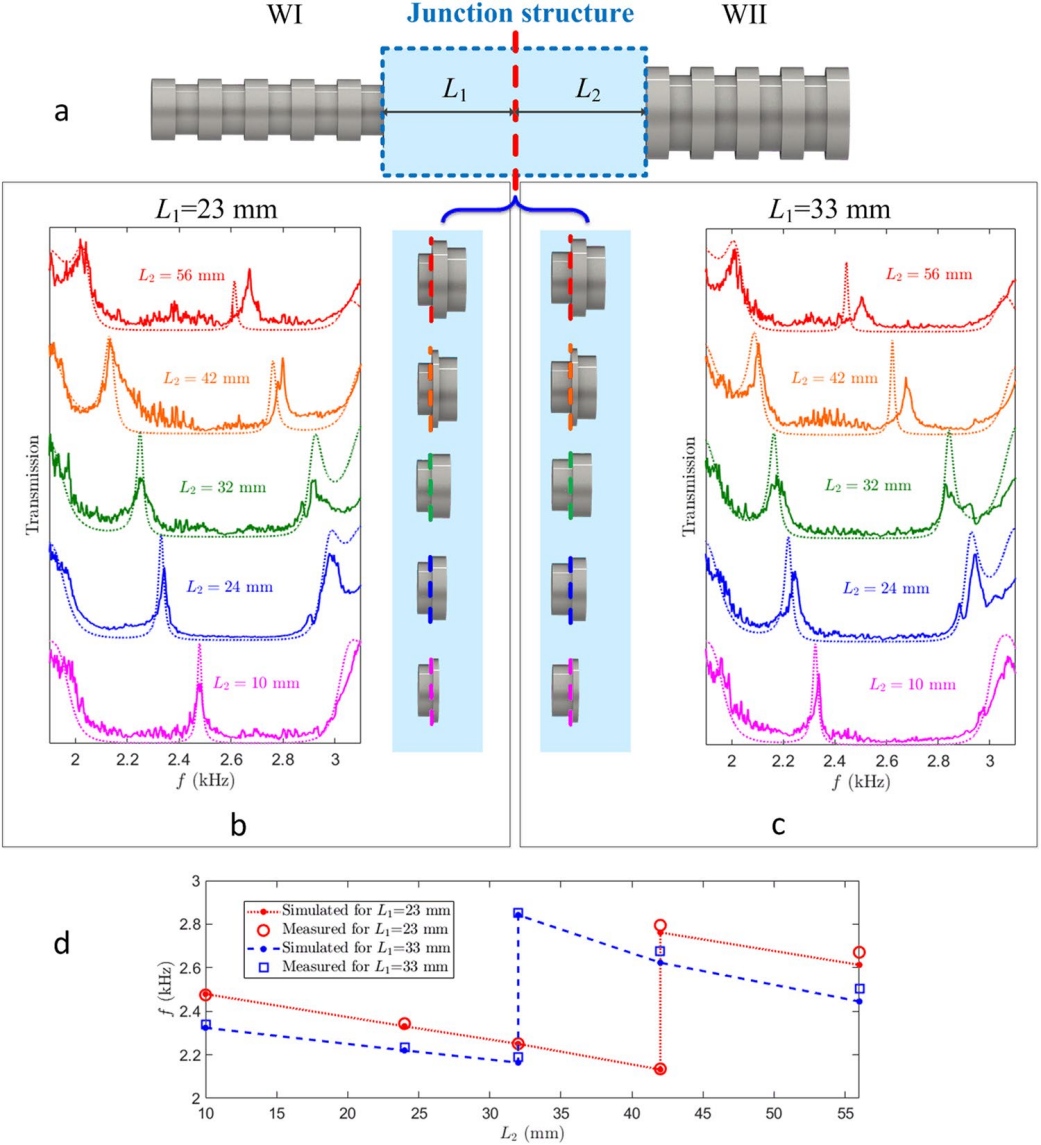
Spatial dependences of transmissions on junction structures. (**a**) Waveguide structure with different heterojunctions in the light blue shadow, which connects Waveguide I and II. The junction structure consists of Waveguide I (WI) with the length *L*_1_ and Waveguide II (WII) with the length *L*_2_. The red dashed line marks the junction interface. (**b,c**) Transmissions of waveguide with different heterojunction structures. In the heterojunctions, the length of WII changes in the order of 10 mm, 24 mm, 32 mm, 42 mm, and 56 mm while that of WI is selected as 23 mm and 33 mm, respectively, in (**b,c**). The altered heterojunctions are also depicted between them and the junction interfaces are marked by the dashed lines in different colours, which are in accordance with these in transmissions. The solid and dotted lines denote the measured and simulated results, respectively. The extraordinary transparency induced by the heterojunction structure shifts in the forbidden band in proper order. (**d**) Peak frequency of the extraordinary transparency for different junction structures. The red circles and blue squares are the measured frequency for junctions with 23 mm and 33 mm long WI, respectively, and the bold dots connected by the dotted and dashed lines show the simulated results as references. It has been found that the extraordinary transmitted peak moves to the low frequency and appears again at the high frequency when it reaches the band edge for the increasing length of WII.

Benefiting from the acoustic scale we considered, the junction structure of the proposed heterojunction waveguide can be tuned and fabricated easily. The acoustic heterojunction waveguide with different heterojunctions is presented in Fig. 2a. For the sake of convenience, we fix the periodic numbers of WI at 5 periods and that of WII at 4.5 periods, and the junction structure consist of WI with the length of *L*_1_ and WII with the length of *L*_2_ as shown in Fig. 2a by the blue rectangular. The red dashed line marks the junction interface, which separates the two waveguides with different numbers of periodic sections. Selecting the lengths of WI and WII in the junction structures can achieve different geometry of heterojunctions and lead to the careful investigation of the contact problems.

To reveal the geometry effects of acoustic heterojunctions, we fabricated two 5-period WIs with *L*_1_ = 23 mm and *L*_1_ = 33 mm, a 4.5-period WII, and five sections of WII with *L*_2_ = 10 mm, 24 mm, 32 mm, 42 mm, and 56 mm. Connecting these structures, we obtained ten waveguides with different heterojunctions as shown between Fig. 2b,c. In these two figures, the experimentally measured and numerically simulated transmissions are depicted by the solid and dotted lines, respectively. The junction interfaces are marked by the dashed lines in different colours and the related transmissions are in the same colour. For *L*_1_ = 23 mm in Fig. 2b, an extraordinary transparency arises near 2.5 kHz when *L*_2_ = 10 mm as shown by the purple lines, and it shifts to the lower frequency when *L*_2_ increases. For *L*_2_ = 42 mm (the orange lines), there are two similar transparent peaks appearing, and it seems that one peak goes into the left band edge and the other comes out from the right band edge. Due to these transmissions, the band edges oscillate more than before. For the larger *L*_2_ = 56 mm (the red lines), there is only one peak left, which is the one shifting out from the high frequency band edge. In general, the extraordinary transparency always moves to the lower frequency range when increasing the length of WII except for a jump from the low frequency to the high one. At this junction structure, the extraordinary transparency moves into the low frequency band edge while a new one comes out from the high frequency edge. Because of the closeness of the two peaks to the band edges, we think that the junction induced transparency disappears at these structure parameters though there are still oscillations. The similar situation has happened for *L*_1_ = 33 mm in Fig. 2c. The shifting property is the same but the transparency disappearing (or the frequency jump) comes for the smaller *L*_2_.

To more clearly identify the junction structure effects, we also present the peak frequency vs the length of WII in Fig. 2d, where the red circles and the blue squares denote the frequencies of the transmitted peaks for *L*_1_ = 23 mm and 33 mm, respectively, and the simulated results are also presented by the bold dots connected by the dotted and dashed lines. It is more obvious that the frequency jumps appear at *L*_2_ = 42 mm and 32 mm, respectively, for short and long WI, though the shift properties to the lower frequency are similar. The junction structure effects appearing in the acoustic system are characterized by the frequency shifting to the lower range with the increasing junction length. It is very intriguing that the peak frequency of the extraordinary transparency moves circularly in the gap and disappears at some junction structure, where the transparencies exhibit the edges of the forbidden band.

### Impedance matching and phase mismatching

To better understand the structure effects in acoustic heterojunction waveguides, we have performed the theoretical and numerical analyses to reveal the mechanism of impedance matching and phase mismatching. The acoustic impedance ratio of a waveguide can be defined as

2 $$Z=\frac{1+R}{1-R},$$

where *R* is the reflection coefficient of the sound pressure at the end. The surface impedance has also been associated with the geometric Zak phase^41,42^ to identify the topological properties of the bulk bands. The topological phase transition can always change the impedance and connecting two structures with different topological properties would result in the arising interface state, where the impedances are opposite in sign. However, the topological theories are very limited to be used in a finite system due to their symmetry requirements. When the working frequency falls into the forbidden band, the impedance ratio should be a pure imaginary number and the impedance matching condition holds for the extraordinary transparency of an acoustic heterojunction waveguide. On the other hand, we set the zero phase points for both WI and WII at the centres of their own narrow pipe sections to achieve the inversion symmetry. At the interface of the two waveguides, the diversions from the centres are defined as the initial phases *φ*_*Ι*_ for WI and *φ*_*ΙΙ*_ for WII. The different junction structures are formed by shifting the phases *φ*_*Ι*_ and *φ*_*ΙΙ*_ independently, and it has been found that the junction structure effects highly rely on the correlation of these two phases.

Based on the coupled mode equations for the acoustic waves in the Bragg gap, we can obtain the reflection coefficients of WI and WII respectively as

3 $$\{\begin{array}{c}{R}_{I}={{\rm{e}}}^{{\rm{i}}(\psi +{\phi }_{I}-\pi /2)}\\ {R}_{II}={{\rm{e}}}^{{\rm{i}}(\psi -{\phi }_{II}+\pi /2)}\end{array},$$

when introducing a real parameter *ψ* for the normalized detuning *δ*_*Ν*_ = cos*ψ* from the Bragg frequency. The impedance ratios are

4 $$\{\begin{array}{c}{Z}_{I}={\rm{i}}\cdot {\rm{ctg}}(\frac{\psi +{\phi }_{I}}{2}-\frac{\pi }{4})\\ {Z}_{II}={\rm{i}}\cdot {\rm{ctg}}(\frac{\psi -{\phi }_{II}}{2}+\frac{\pi }{4})\end{array}.$$

**Figure 3:**
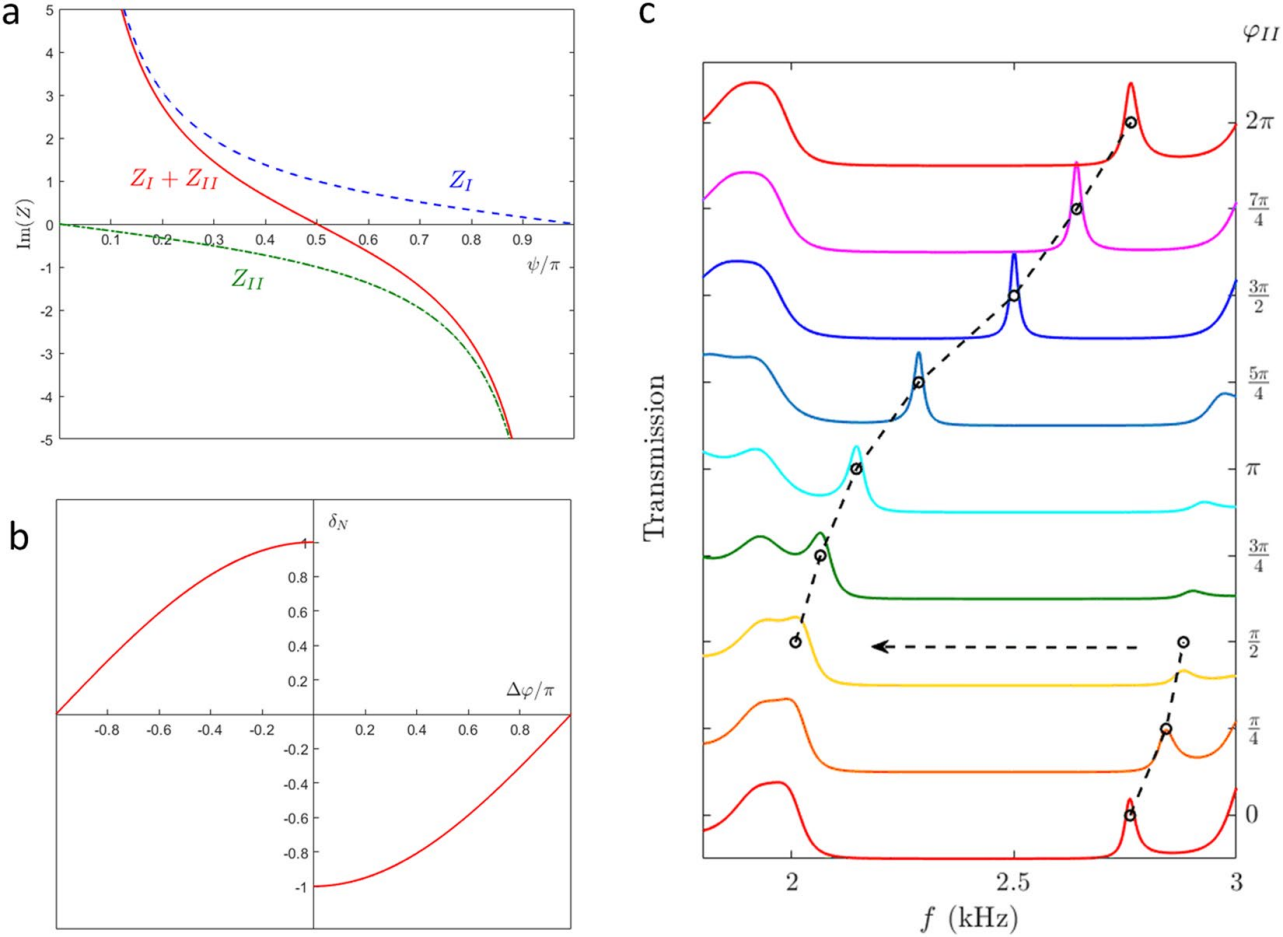
Transparency appearing and disappearing. (**a**) Acoustic impedance ratio in the forbidden band. The blue dashed and green dash-dot lines denote the reflection impedance ratios at the interfaces of WI and WII, respectively. The red solid line represents their summation and the zero point at *ψ* = 0.5*π* indicates the extraordinary transparency appearing. (**b**) Transparency frequency shift from the Bragg frequency vs the phase difference between WI and WII. The frequency detuning *δ*_*N*_ is normalized by the half width of the forbidden band. Thus, *δ*_*N*_ = ±1 indicates that the transmitted peak falls at the edge of the forbidden band and the extraordinary transparency disappears when the phase matching Δ*φ* = 0. Only when the phases mismatch, the frequency falls in the forbidden band and we can observe the extraordinary transparency. (**c**) Transmission for the waveguides with different junction structures, which are formed by changing the phase of WII *φ*_*ΙΙ*_ and fixing that of WI *φ*_*Ι*_ at *π*/2. The circles connected by the dashed lines indicate the peak frequency of the transparency shifting to the high frequency when increasing *φ*_*ΙΙ*_ and a frequency jump arising when the phases match at *φ*_*ΙΙ*_ = *φ*_*Ι*_ = *π*/2.

The imaginary parts of the impedance ratios and their summation for varying frequency detuning are presented in Fig. 3a for *φ*_*Ι*_ = *π*/2 and *φ*_*ΙΙ*_ = −*π*/2 according to the waveguide in Fig. 1a. We can find that *Z*_*I*_ and *Z*_*II*_ are totally positive and negative in the range of frequency detuning and there is only one zero for their summation (the red solid line), where the impedance matching condition holds at *ψ* = *π*/2. Then, the zero frequency detuning means that the extraordinary transparency appears in the middle of the Bragg gap, which has been observed in the experiment as shown in Fig. 1c.

Solving the impedance matching equation *Z*_*I*_ + *Z*_*II*_ = 0 yields *ψ* = Δ*φ*/2+*nπ* with *n* = 0, 1, where the relative phase shifting Δ*φ* = *φ*_*ΙΙ*_ −*φ*_*Ι*_ . Then we can obtain the frequency detuning *δ*_*Ν*_ = cos(Δ*φ*/2 + *nπ*). Fixing *φ*_*Ι*_ and increasing *φ*_*ΙΙ*_ mean shortening the junction structure between WI and WII, which results in the high frequency shifting of the transparency. Therefore, only the monotone increasing solutions are left as shown in Fig. 3b. There is a frequency jump at *φ*_*Ι*_ = *φ*_*ΙΙ*_, which have been obtained in the experiments. It turns out to be that the phase matching junction structure causes the transparency disappearing while the heterojunction induced transparency can be only attributed to the structure phase mismatching. In our experiments, *φ*_*Ι*_ ≈ 7/6*π* and 3/2*π*, then the transparencies disappear at *φ*_*ΙΙ*_ ≈ 7/6*π* (*L*_2_ = 42 mm) and 3/2*π* (*L*_2_ = 32 mm), respectively. The further simulated results confirm the theoretical findings on the phase mismatching conditions for the extraordinary transparency appearing, and a typical example for *φ*_*Ι*_ = *π*/2 is shown in Fig. 3c.

## Discussion

We have found the heterojunction proximity effects experimentally and theoretically in an elaborated acoustic heterojunction waveguide. The fabrication of the acoustic heterojunction, the arising extraordinary transparency, and its dependence on the junction structures have been demonstrated. The acoustic heterojunction consists of two different periodic waveguides with the similar bandgap analogous to those in semiconductors and the energy can be localized in its interface at some special frequencies, where an extraordinary transparency has been observed experimentally. It has been found that the transparency frequency highly relies on the junction structures. Changing the junction structure yields the frequency circularly moving in the gap and the extraordinary transparency disappears for a special junction. The further theoretical analysis on the acoustic impedance ratios reveals the transparency appearing conditions of impedance matching and phase mismatching. It is also confirmed by the experiments and numerical simulations that the extraordinary transparency appears when the acoustic impedances match at the interface and the phases of the two waveguides are different. If we connect two waveguides with the same phase, there will be a frequency jump and the transparency disappears. The junction structure effects found in acoustic heterojunctions provide opportunities to understand the geometry or contact problems of heterojunctions. It is very convenient to manipulate the heterojunction performance by the initial phase of wall corrugations beyond the momentum space phases. The physical aspects on acoustic heterojunctions can also be generalized to other systems and benefit the innovations on functional and controllable devices in various applications, such as underwater sound control, communications, terahertz technologies, phononics, and photonics.

## Methods

### Structure preparation

We fabricated the acoustic heterojunction using stainless steel pipes with different inner diameters. For Waveguide I, we first lathed the thick pipe with 100 mm outer and 72 mm inner diameters into a 297-mm-long piece. Second, we lathed the inner diameter corrugation with the turning machine by widening the inner diameter to 88 mm every 33 mm long. Finally, we processed the thread at both ends of Waveguide I. Similar to Waveguide I, we lathed the pipe with 140 mm outer and 108 mm inner diameters into a 288-mm-long piece of Waveguide II. After lathing the inner diameter corrugation by widening the diameter to 132 mm every 32 mm long, we also processed the thread at both ends. The acoustic heterojunction was completed by tightening the thread at the ends. Furthermore, the junction structures were also lathed with the turning machine in the similar way.

### Experimental configuration and measurements

In the experiments, a National Instruments card PCI-4461 generated a harmonic signal, which was magnified by an amplifier and used to excite the coaxial monitor speaker. The monochromic sound from the speaker was transmitted into a 500-mm-long straight waveguide with an inner diameter of 88 mm. The straight waveguide was used to reform the acoustic wave into the fundamental mode. Another straight duct was used at the other end of the acoustic heterojunction to regulate the output impedance. A microphone (G.R.A.S. 1/4′′ free-field microphone 46BE) in a 2-m-long pipe was used to measure sound pressure and it was held by a 3-dimensional motorized translation stage, which afforded the 3-dimensional movements of the microphone throughout the structure. The PCI-4461 card sampled and recorded the received signals at 200 kHz. By using a tone-extraction analysis, we identified the frequency and amplitude of the obtained sound pressure.

### Numerical calculations

The numerical simulations were performed by COMSOL Multiphysics in this work. In the simulations, we set the hard-boundary sound conditions to the outer boundaries of the acoustic heterojunction, selected the air with the density 1.25 kg/m^3^ and the sound speed 343 m/s in the waveguide, and assigned the plane-wave radiation boundary conditions to the inlet and outlet. By using the boundary integration tool, we calculated the transmittance with the incident and transmitted sound powers.
